# Evaluation of a family-oriented antenatal group educational program in rural Tanzania: a pre-test/post-test study

**DOI:** 10.1186/s12978-018-0562-z

**Published:** 2018-06-28

**Authors:** Yoko Shimpuku, Frida E. Madeni, Shigeko Horiuchi, Kazumi Kubota, Sebalda C. Leshabari

**Affiliations:** 1 Human Health Sciences, Graduate School of Medicine, Kyoto University, 53 Shogoin-kawahara-cho, Sakyo-ku, Kyoto, 606-8507 Japan; 2 Magunga District Hospital, P. O. Box 430, Old-Korogwe, Tanga, Tanzania; 3 Department of Biostatistics, Yokohama City University School of Medicine, 3-9 Fukuura, Kanazawa-ku, Yokohama, 236-0004 Japan; 4 School of Nursing, Muhimbili University of Health and Allied Sciences, P. O. Box 65169, Dar es Salaam, Tanzania

**Keywords:** Pregnancy, Childbirth, Family support, Birth preparedness, Antenatal education, Africa

## Abstract

**Background:**

To increase births attended by skilled birth attendants in Tanzania, studies have identified the need for involvement of the whole family in pregnancy and childbirth education. This study aimed to develop, implement, and evaluate a *family-oriented antenatal group educational program* to promote healthy pregnancy and family involvement in rural Tanzania.

**Methods:**

This was a quasi-experimental 1 group pre-test/post-test study with antenatal education provided to pregnant women and their families in rural Tanzania. Before and after the educational program, the pre-test/post-test study was conducted using a 34-item Birth Preparedness Questionnaire. Acceptability of the educational program was qualitatively assessed.

**Results:**

One-hundred and thirty-eight participants (42 pregnant women, 96 family members) attended the educational program, answered the questionnaire, and participated in the feasibility inquiry. The mean knowledge scores significantly increased between the pre-test and the post-test, 7.92 and 8.33, respectively (*p* = 0.001). For both pregnant women and family members, the educational program improved *Family Support* (*p* = 0.001 and *p* = 0.000) and *Preparation of Money and Food* (*p* = 0.000 and *p* = 0.000). For family members, *the scores for Birth Preparedness* (*p* = 0.006) and *Avoidance of Medical Intervention* (reversed item) (*p* = 0.002) significantly increased. Despite the educational program, the score for *Home-based Value* (reversed item) (*p* = 0.022) and *References of SBA* (*p* = 0.049) decreased in pregnant women. Through group discussions, favorable comments about the program and materials were received. The comments of the husbands reflected their better understanding and appreciation of their role in supporting their wives during the antenatal period.

**Conclusions:**

The *family-oriented antenatal group educational program* has potential to increase knowledge, birth preparedness, and awareness of the need for family support among pregnant women and their families in rural Tanzania. As the contents of the program can be taught easily by reading the picture drama, lay personnel, such as community health workers or traditional birth attendants, can use it in villages. Further development of the Birth Preparedness Questionnaire is necessary to strengthen the involved factors. A larger scale study with a more robust Birth Preparedness Questionnaire and documentation of skilled care use is needed for the next step.

**Trial registration:**

No.2013–273-NA-2013-101. Registered 12 August 2013.

## Plain English summary

One of the biggest challenges facing Tanzania is the large number of women who die from pregnancy and childbirth, reflecting the need for better antenatal education. In response to this need, our group of Japanese and Tanzanian midwives developed an antenatal group educational program for teaching the importance of birth preparedness and family involvement in pregnancy in rural Tanzania. For the educational material, we developed a picture drama which compared the stories of two women who had different birth preparations and family involvement. We provided the program to pregnant women and their families in Kiswahili, and asked them to fill questionnaires before and after the program and to join the discussion regarding their thoughts about the program. There were 138 participants who attended the program, answered the questionnaires, and participated in the discussion. Their knowledge of danger signs of pregnancy increased. Moreover, their perceptions toward family support and preparation of money and food improved. Family members showed better preparedness for birth and less avoidance of medical intervention. The comments about the program and materials were favorable. The husbands’ comments reflected better understanding and appreciation of their role in supporting their wives during the antenatal period. Taken together, the antenatal group educational program increased knowledge, birth preparedness, and awareness of the need for family involvement and support among pregnant women and their families in rural Tanzania. Educational programs using picture dramas could add an acceptable component for increasing birth preparedness among pregnant women and their families especially in rural areas.

## Background

In 2015, the Sustainable Development Goals of the United Nations were published on the basis of the achievement of the Millennium Development Goals (MDGs) [1–3]. According to the MDGs Report, greater effort is needed to reduce the global burden of maternal mortality [1]. Although a 45% reduction of maternal mortality in developing countries is a significant achievement, still about 289,000 women died in 2013 from causes related to pregnancy and childbirth [1]. World Health Organization (WHO), ICM (International Confederations of Midwives), and FIGO (International Federation of Gynecology and Obstetrics) have suggested that to reduce maternal mortality, skilled birth attendants (SBAs), namely, midwives, nurses, or doctors, should assist all births [4]. In rural areas of Tanzania, however, only 54% of all deliveries were reportedly conducted by SBAs [5]. Thus, nearly half of rural women still either chose not to use SBAs or had no access to SBAs while giving birth.

Researchers have indicated that Birth Preparedness and Complication Readiness (BP/CR) could be a key factor in influencing the choice of birthplace with SBAs [6–8]. In the WHO publication of *Birth and Emergency Preparedness in Antenatal Care* [9], 9 birth preparation components were identified: (1) the desired place of birth; (2) the preferred birth attendant; (3) the location of the closest appropriate care facility; (4) money for birth-related and emergency expenses; (5) a birth companion; (6) support in looking after the home and children while the woman is away; (7) transport to a health facility for the birth; (8) transport in the case of an obstetric emergency; and (9) identification of compatible blood donors in case of emergency. Despite the importance of BP/CR to promote SBAs, several reports have suggested a low level of BP/CR among women in African settings [10–13].

To increase BP/CR, 2 review studies have shown the accumulated findings of interventions. One study reviewed 58 articles and identified that community-based information, education, and communication interventions, which taught women when to reach out for assistance, increased awareness and knowledge of the danger signs of pregnancy complications [14]. This increase of women’s awareness and knowledge has resulted in an increase in the utilization of health facility delivery services [14]. Another study using systematic review and meta-analysis found that exposure to BP/CR interventions was associated with a significant reduction of 18% in neonatal mortality risk (12 studies, RR = 0.82; 95% CI: 0.74, 0.91) and a nonsignificant reduction of 28% in maternal mortality risk (7 studies, RR = 0.76; 95% CI: 0.69, 0.85) [15]. Both home visits and community-based women’s group sessions have been reported to potentially reduce the risk of neonatal mortality; however, their effects on maternal health have not been fully clarified. Moreover, home-based individual counseling has been found to be more personalized and appropriate for developing mothers’ personal knowledge and skills [15]. However, other studies have revealed the lack of decision-making power of women within their family with regard to the referral and place of birth [16–18].

Intra-family or extended family decision-making also influences women’s choices for childbirth [19]. The values and opinions of the husband, mother-in-law, mother, traditional birth attendant, other family members, and community members have been shown to have more influence in decisions regarding the birth place than the pregnant woman’s input [19]. Implementation of the 9 BP/CR components requires family support. This implies that the decision-maker of the household, mostly the husband, must agree to provide the financial support for transportation and funds for emergency support, and the family or extended family should provide for child care, arrange for possible transport, be prepared to donate blood if necessary, and come to an agreement regarding the location for childbirth [20]. Even though husbands were typically the decision-makers regarding the place of delivery, they were rarely encouraged to attend antenatal sessions [21, 22]. August et al. [20] specifically interviewed husbands regarding their understanding of BP/CR and found them lacking particularly in the area of identifying an SBA. Therefore, community-based activities were still needed to promote family involvement because in these traditional settings the locus of decision-making was more community-based than individual-based [15]. WHO [16] recommended using BP/CR education and discussion with community participation, particularly the involvement of the male partner and other householder decision-makers.

It was found in Uganda that women who prepared for birth in consultation with their family members were more likely to give birth with the help of SBAs than women who prepared for birth by themselves [7]. A previous study investigating the partners’ influence on women delivering at a health facility in Tanzania found that the agreements of partners on the importance of delivering in a health facility and on doctors having better skills than traditional birth attendants were associated with delivery in a health facility [21].

For over a decade, studies conducted in Tanzania have identified the need for involvement of the whole family in pregnancy and childbirth education [23–26]. Shimpuku et al. [26] emphasized the importance of family involvement in pregnancy and childbirth because it improved the quality of care and women’s birth experiences.

Therefore, the role of the family in decision-making is a crucial issue for reducing maternal mortality, with the place of birth being an important component. However, there are only few studies that have examined family-based education. We therefore performed the present study to evaluate our recently developed *family-oriented antenatal group educational program* for pregnant women and their family members in rural Tanzania.

## Methods

### Study design

This was a quasi-experimental, 1 group pre-test/post-test study that included a qualitative component addressing feasibility. The *family-oriented antenatal group educational program* was provided to increase birth preparedness.

### Setting

We conducted this study in Korogwe district, which is located in the center of the Tanga Region of North Eastern Tanzania. Korogwe has a total area of 3756 sq. km with 132 villages [27]. The main economic activities include agriculture and horticulture involving the natural resources of forests and game parks. The Korogwe district is predominately rural with a large population of 175,339 in reference to an urban area of 43,510. The Korogwe district has 1 public hospital and 2 private hospitals, 3 health centers, and 59 dispensaries. According to Demographic Health Survey 2 (DHS2) in 2016, delivery at health facilities 6969, deliveries with Traditional Birth Attendant (TBA) 259, home deliveries without TBA 151, birth before arrival 118 [28].

### Sampling method and sample size

With the support of a local collaborator, we purposefully selected 3 mountain villages where the nearest health center was located at least 5 km away, and the majority of women deliver at home. As the villages were located in the mountain, women in these villages must walk unpaved mountain roads to reach the nearest health center. The roads become completely dark after sunset; therefore, preparation of transportation and financial support was necessary to reach skilled care because contractions could start unpredictably, even at night.

We aimed to recruit a total sample size of 100 participants (50 pregnant women and 50 family members) to meet the assumption of a normal distribution [29]. Hence, 100 participants were expected to attend the program and pre-test/post-test. The inclusion criteria for women were as follows: currently pregnant with no severe physical or psychological illness. We did not exclude women based on gestational weeks or number of pregnancies. The criteria for family members were as follows: 16 years old or older, living with or near the pregnant woman, and defined as “family” by the pregnant woman (regardless of their blood or marital relationship). Participants need to be able to read Kiswahili to complete the self-administered questionnaire. If they need assistance in answering the questionnaire, a research assistant helped for reading and marking the answer.

### Recruitment and data collection process

We requested the village leaders to inform their constituents about our research activities and to ask pregnant women and their families to gather in a school or a church on a specified date and time. When we arrived in the village, pregnant women and their families gathered at the specified place. After explaining the details of our research, they were asked if they agreed to participate in the study. Because of the interest of those who gathered in the study, all agreed to participate and stayed to receive the educational program and pre-test/post-test. This data collection process was repeated in 2 more villages.

### Educational program

We developed the *family-oriented antenatal group educational program* to be culturally relevant. The program lasted for approximately 2 h and included a pre-test/post-test, picture drama, and discussion. In the educational program, pregnant women and their families were taught the importance of birth preparation to increase the safety of childbirth, namely, survival of pregnant women and their babies. The program aimed to inculcate the following ideas: (1) preparation for childbirth is important to enable women to receive health care when necessary, (2) families are encouraged to support the women’s choice when deciding their birth place, and (3) women are advised to give birth with an SBA. We created a story of women’s birth experiences based on a qualitative study at a rural hospital in Tanzania [26]. The story included health promotion concepts such as family decision-making processes, and family support according to the *Integrated Management of Pregnancy and Childbirth* [9].

At the beginning of our *family-oriented antenatal group educational program*, the participants were asked to answer the Birth Preparedness Questionnaire (BPQ) pre-test. The second author (FM), a female Tanzanian researcher, who has a master’s degree with knowledge of local customs, shared a story using a picture drama. The picture drama depicted 2 women who had very different birth experiences, one with and one without proper birth preparation. The first woman had a very supportive family. She attended antenatal visits regularly. At the visits, a midwife provided information about nutrition, danger signs during pregnancy, risks of some traditional herbs, potential problems of the mother and baby, and necessary preparations including money, transportation, birth companions, and blood donors in case of emergency. As a result, this first woman who had properly prepared for birth with her family had a normal delivery of a healthy baby. The second woman had a family who strongly believed in home birth and did not allow her to go to antenatal visits. Because the family did not prepare anything, when the woman’s labor become obstructed, they delayed leaving for the hospital and consequently, the family lost both the mother and the baby. The pictures clearly depicted the story and illustrated Tanzanian housing and clothes (*kanga*) to enable the participants to easily identify with the story.

In community settings, utilization of picture drama was found to be educationally effective. An increase in knowledge was found when providing reproductive health education using picture dramas to adolescent boys and girls in Tanzania [30]. The pictures made it easier for those who were not strong in health literacy to understand the contents. All important information was written on the backside of the pictures. This enabled the presenter to be consistent and provide comprehensive information. After the picture drama and the presenter’s explanation, the participants were asked to answer the same BPQ for the post-test and then to evaluate the contents and effectiveness of the *antenatal group educational program*.

### Measurements

Demographic items included age, marital status, education, occupation, daily monetary use, household assets, ethnic group, distance from health care facility, experience in losing a family member owing to pregnancy problems, and obstetric history (only pregnant women).

#### Birth preparedness questionnaire

The BPQ is a 34-item self-administered questionnaire consisting of a knowledge test and a BP/CR assessment that was used for both the pre-test ad the post-test. In the development of the questionnaire, the authors utilized Ajzen’s theory [31] as a guide. The theory explains that intentions to perform various behaviors can be predicted from the following 3 perceptional components: 1) perceived behavioral control, 2) attitudes toward the behavior, and 3) subjective norms. The authors chose this theory to frame items related to those 3 psychological factors that may influence intention to have an SBA.

Knowledge items were added to the questionnaire because previous studies have indicated that adequate knowledge of danger signs was associated with more SBA-attended births [32, 33]. We developed a self-administered 10-item knowledge test about safe pregnancy and danger signs based on the 9 components of *Integrated Management of Pregnancy and Childbirth* [9]. The items are presented as binary *yes/no* responses. Another 24-item BP/CR assessment test was developed to assess BP/CR and the items were related to psychological values and beliefs. These 24 items were rated using a 3-point Likert scale indicating (1) *disagree,* (2) *neither disagree nor agree*, or (3) *agree.*

The questionnaire was first developed in English and then translated into Kiswahili, which is a more familiar language to most Tanzanians. The questionnaire was translated by the second author (FM) who is bilingual in Kiswahili and English, from the same district, and experienced in communicating with rural Tanzanian women. Initially, to increase face validity, 10 rural women were interviewed regarding their answers for the knowledge test and BP/CR assessment. They were asked to provide their opinions regarding any unclear or confusing questions. Thereafter, the knowledge test and BP/CR assessment were revised according to the opinions of the interviewed women and finalized for this study.

As it was the first time to introduce the program to Tanzania, a feasibility inquiry was conducted in the form of a group discussion that included all the women and their family members attending the program, followed by completion of a post-test questionnaire. This feasibility inquiry was conducted to initially determine if the delivery of the program was acceptable to the participants. The participants responded to open-ended questions to assess the feasibility of the study. The following questions were included: 1) “How did you feel about the contents of the picture drama? Did you understand it well? If not, what was difficult to understand?” 2) “Are the contents of the picture drama appropriate for educating rural women and their family members?” 3) “How can we improve the contents of the picture drama?”. While observing the discussions, field notes were taken and data were transcribed in Kiswahili and then translated into English. Thematic content analysis [34] was used based on the predetermined concepts of acceptability, demand, implementation, practicality, and limited efficacy [35]. The field notes were carefully read and similar ideas were extracted, then the major ideas were discussed. Thus, the major findings were organized into 5 categories.

### Ethics approval and consent to participate

The study was conducted based on the principles of ethics such as harmlessness, being voluntarily, anonymity, and protection of privacy and personal information. We explained these principles during recruitment along with the purpose, methods, and ethical considerations. We asked each participant if they agreed to participate in the study and only those who agreed were included in the study. We obtained verbal consent because the information gathered was unidentifiable and the risk from this study was minimal. Ethical clearance and permissions were obtained from the 1) Research Ethics Committee of St. Luke's International University (14–040); 2) Director of the Korogwe District Council, 3) National Institute for Medical Research, Tanzania (NIMR/HQ/R.8/Vol.IX/1604), and 4) Tanzania Commission for Science and Technology (No. 2013–273-NA-2013-101).

### Data analysis

SPSS ver. 22.0 was used for descriptive analysis, correlations, exploratory factor analysis using the maximum likelihood method and promax rotation to validate the factor structure. Exploratory factor analysis was conducted using the 28 original BP/CR items to determine whether measures of the construct were consistent with authors’ understanding of the nature of that construct. Before the analysis, the answers for all the reverse items were inversed (from 1 to 3 and from 3 to 1). Four items were excluded during the analysis because of low correlations. The final factor analysis included the remaining 24 items. As a result, 7 factors were identified: *Home-based Value* (Factor I, 7 items, Cronbach’s alpha = 0.846), *Birth Preparedness* (Factor II, 5 items, Cronbach’s alpha = 0.691), *Family Support* (Factor III, 4 items, Cronbach’s alpha = 0.646), *Avoidance of Medical Intervention* (Factor IV, 2 items, Cronbach’s alpha = 0.548), *Preparation of Money and Food* (Factor V, 2 items, Cronbach’s alpha = 0.615), *Preference of SBA* (Factor VI, 2 items, Cronbach’s alpha = 0.472), *Pregnant Women’s Workload* (Factor VII, 2 items, Cronbach’s alpha = 0.337). Table 1 shows the results of factor analysis. Wilcoxon signed-rank test was used to determine any significant difference in the BP/CR scores before and after the educational program. For the feasibility inquiry, the qualitative data were analyzed using thematic content analysis.

**Table 1:** Exploratory factor analysis Birth Preparedness Questionnaire using maximum likelihood method and promax rotation among 138 rural adults in Tanzania

Item#	Items	I	II	III	IV	V	VI	VII
Factor 1: Home-based value								
Q36	I plan to give birth at home. (reverse item)	**.839**	−.026	.029	.058	−.019	−.051	.091
Q25	I want (I want her) to give birth at home with family because we do not know people in health care facilities (reverse item)	**.799**	.025	.040	.049	.158	−.299	−.058
Q38	God will help me, so I don’t plan anything for childbirth. (reverse item)	**.755**	.153	.122	.201	−.211	−.183	−.165
Q29	A pregnant woman should stay at home to give birth if no one else takes care of the children in the family. (reverse item)	**.621**	−.179	−.160	.030	.104	.087	.284
Q28	A woman should give birth at home if her husband does not allow her to go to a health center. (reverse item)	**.593**	−.095	−.295	−.067	.071	.341	−.038
Q24	I like to give birth with traditional birth attendants because they are kinder than nurses at the hospital. (reverse item)	**.554**	.050	.129	.434	.012	−.048	.156
Q31	A baby belongs to the family, so woman should follow their family’s wishes about where to give birth. (reverse item)	**.474**	−.004	−.517	−.139	.000	.152	−.131
Factor 2: Birth preparedness								
Q18	My family members think it is okay for me to access health care.	.060	**.754**	.121	.113	−.050	.040	−.130
Q13	I have someone who can go with me when going to a health care facility for birth	−.083	**.667**	.021	.206	.034	.223	.155
Q12	I have someone who can take over my family responsibilities when I need to go to a health center.	−.083	**.625**	−.123	.028	.353	−.042	.142
Q15	I know where to find a health care facility for delivery and emergencies.	.154	**.607**	−.128	−.361	.100	−.183	.134
Q14	I can identify where a trained health care provider is to help me.	−.153	**.574**	−.205	−.330	−.173	.088	−.053
Factor 3: Family support								
Q34	I will prepare for childbirth with my family	.056	−.139	**.770**	−.056	.280	−.107	−.078
Q30	A woman should discuss her birth with her husband and other family members.	−.081	−.047	**.746**	−.029	.089	.003	.158
Q22	It is better to go to a health center for deliveries with family members because of their support.	.138	.058	**.507**	−.411	−.314	.333	.101
Q35	I am going to give birth at a health care facility	.051	.150	**.461**	.155	.074	.287	−.247
Factor 4: Avoidance of medical intervention								
Q23	I would not go to a health center because I do not want a C-section or a vaginal cut. (reverse item)	.180	.113	−.020	**.771**	−.096	−.084	−.114
Q20	It is okay to give birth at home when a pregnant women and her baby have no problems. (reverse item)	.138	−.122	−.027	**.507**	−.095	.277	.073
Factor 5: Provision of money and food								
Q11	I have saved enough money to reach a health facility for birth.	.115	.073	.111	−.157	**.791**	.091	−.309
Q32	I plan to eat healthy during my pregnancy.	−.052	−.014	.267	.010	**.688**	.278	.076
Factor 6: Preference of SBA								
Q37	I will visit a health centre if there is a problem for my baby or me after delivery.	−.171	.020	−.026	.003	.187	**.815**	−.165
Q26	I want to be with doctors and nurses during childbirth because they are the experts.	.108	.276	.022	.089	.281	**.409**	.121
Factor 7: Pregnant women’s workload								
Q21	Women should rest and should not work hard during their pregnancy.	.082	.084	.215	−.413	.033	−.153	**.746**
Q27	A woman should work hard for her family during pregnancy. (reverse item)	−.002	.054	−.125	.245	−.290	−.074	**.723**

Component values are captured in bold

## Results

### Demographic and obstetric characteristics

There were a total of 139 prospective participants from 3 villages (42 pregnant women and 97 family members). One participant did not complete the questionnaire and was therefore excluded. Hence, 138 participants (42 pregnant women and 96 family members) were included for the analysis. Table 2 shows a summary of the participants’ characteristics. Although the group included both pregnant women and their family members, there was no significant difference in education, occupation, daily expenses, or household assets. There was a significant difference in the mean age (*p* < 0.001). Among the 42 pregnant women, 8 (19%) were primipara and 34 were multipara (*M* = 1.45, *SD* 1.194, range 0–5). For antenatal visits, 17 (40.5%) attended once and 23 (54.8%) attended twice (2 had missing data). There were 24 multiparas who had given birth at a health facility, one of whom had caesarean section. None had lost a baby during pregnancy or childbirth. All the participants attended the *antenatal group educational program* and the *feasibility inquiry*.

**Table 2:** Comparison of the sociodemographic characteristics of the participants

	Pregnant women (*N* = 42)		Family (*N* = 96)		*p-value*
	Mean (SD)	n (%)	Mean (SD)	n (%)	
Age	27.67		35.29		< 0.001
Educational level					0.148
Lower than secondary level		34 (82.9)		62 (73.8)	
Secondary level and above		7 (17.1)		22 (26.2)	
Missing data		1 (2.4)		12 (12.5)	
Occupation					0.203
Farmer		33 (80.5)		63 (73.3)	
Housewife/student		8 (19.5)		23 (26.7)	
Missing data		1 (2.4)		10 (10.4)	
Daily expense					0.662
< 1000 TSH		18 (42.9)		39 (47.0)	
1000–5000 TSH		21 (50.0)		32 (38.6)	
≥ 5000 TSH		3 (7.1)		12 (14.4)	
Missing data		0		13 (13.5)	
Household assets ownership					0.83
Low (0–1)		38 (90.5)		70 (75.3)	
High (2+)		4 (9.5)		23 (24.7)	
Missing data		0		3 (3.1)	

### Pre-test and post-test differences in knowledge

**Fig. 1:**
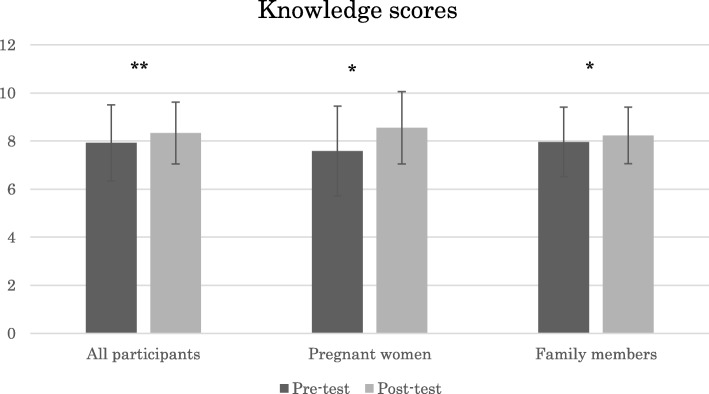
Knowledge scores of all the participants, pregnant women, and family members before and after the *family-oriented antenatal group educational program* in rural Tanzania

Figure 1 shows that the mean knowledge score of all the participants significantly increased between pre-test and post-test (*p* = 0.001). After the education, both pregnant women and family members shored a significantly higher mean knowledge scores (*p* = 0.011 and *p* = 0.020, respectively).

### Pre-test/post-test differences in BPQ

There were significant differences between pre-test and post-test both in pregnant women and family members in *Family Support* (*p* = 0.001 and *p* = 0.000, respectively) and *Preparation of Money and Food* (*p* = 0.000 and *p* = 0.000, respectively). Table 3 shows the mean score and standard deviation of the 7 factors between pregnant women and family members for the pre-test and post-test. There was a significant decrease in two reversed items of *Home-based Value* and *Preference of SBA* (*p* = 0.022 and *p* = 0.049, respectively) among only pregnant women. There were significant differences in *Birth Preparedness* and *Avoidance of Medical Intervention* (*p* = 0.006 and *p* = 0.002, respectively) among only family members.

**Table 3:** Comparison of pre-test/post-test Birth Preparedness Questionnaire scores among pregnant women and family members

	Pregnant women (*n* = 42)			Family members (*n* = 96)		
	Pre	Post	*p*	Pre	Post	*p*
1) Home-based Value	17.32 (3.49)	16.33 (3.88)	0.022*	16.33 (3.88)	16.24 (3.51)	0.312
2) Birth Preparedness	14.13 (1.07)	14.38 (1.01)	0.135	14.38 (1.01)	13.99 (1.76)	0.006**
3) Family Support	10.02 (1.76)	11.18 (1.58)	0.001**	11.18 (1.58)	10.95 (1.52)	0.000**
4) Avoidance of Medical Intervention	3.92 (1.61)	4.25 (1.56)	0.078	4.25 (1.56)	4.26 (1.37)	0.002**
5) Preparation of Money and Food	4.77 (1.28)	5.68 (0.75)	0.000**	5.68 (0.75)	5.71 (0.66)	0.000**
6) Preference of SBA	5.78 (0.70)	5.51 (0.93)	0.049*	5.51 (0.93)	5.53 (0.77)	0.105
7) Pregnant Women’s Workload	4.80 (1.31)	5.08 (1.11)	0.108	5.08 (1.11)	4.64 (1.10)	0.215

*p < 0.05, **p < 0.01

Note: The Wilcoxon signed-rank test. The *p*-value is asymptotic significance (two-tailed)

### Correlation among BPQ factors

Among the 7 factors, *Home-based Value* was positively associated with *Avoidance of Medical Intervention* (*r* = 0.55, *p* = 0.000) and *Pregnant Women’s Workload* (*r* = 0.22, *p* = 0.011) and negatively associated with *Family Support* (*r* = − 0.34, *p* = 0.000) and *Preparation of Money and Food* (*r* = − 0.26, *p* = 0.000). *Birth Preparedness* was positively associated with *Family Support* (*r* = 0.26, *p* = 0.002), *Preparation of Money and Food* (*r* = − 0.28, *p* = 0.001), and *Preference of SBA* (*r* = 0.24, *p* = 0.05), and negatively associated with *Avoidance of Medical Intervention* (*r* = − 0.29, *p* = 0.001). *Family Support* was positively associated with *Preparation of Money and Food* (*r* = 0.41, *p* = 0.000) and *Preference of SBA* (r = 0.26, *p* = 0.003) and negatively associated with *Avoidance of Medical Intervention* (*r* = − 0.27, *p* = 0.002). *Avoidance of Medical Intervention* was negatively associated with *Preparation of Money and Food* (*r* = − 0.25, *p* = 0.003). *Preparation of Money and Food* was positively associated with *Preference of SBA* (*r* = 0.31, *p* = 0.000).

### Feasibility inquiry

Bowen et al. [35] suggested 8 general areas of focus for a feasibility study: *acceptability*, *demand*, *implementation*, *practicality*, *adaptation*, *integration*, *expansion*, and *limited efficacy*. Within these areas, this study focused on acceptability, demand, implementation, practicality, and limited efficacy, which fit with the preliminary stage of this study. The details and outcomes are described in Table 4.

**Table 4:** Observations and qualitative data for the feasibility inquiry

Focus areas	Questions in feasibility inquiry^a^	Study outcomes
Acceptability	To what extent is a new idea, program, process or measure judged as suitable, satisfying, or attractive to program deliverers? To program recipients?	“The picture is good and enables us to easily understand.” “We are very happy to learn many things, no one feels bad. Everybody is happy.”
Demand	To what extent is a new idea, program, process, or measure likely to be used (i.e., how much demand is likely to exist?)	“This type of education should be given to all Tanzanian people, pregnant women and their families.”
Implementation	To what extent can a new idea, program, process, or measure be successfully delivered to intended participants in some defined, but not fully controlled, context?	It takes more time to complete the self-administered questionnaire than what was planned by the researchers.
Practicality	To what extent can an idea, program, process, or measure be carried out with intended participants using existing means, resources, and circumstances and without outside intervention?	The collaborator can conduct the intervention, but more local nurses or community health workers need to be educated to implement the intervention and expand the research.
Limited efficacy	Does the new idea, program, process, or measure show promise of being successful with the intended population, even in a highly controlled setting?	The scores of the measure showed some significant differences before and after the intervention with the limited convenient sample. More changes are expected.

^a^Referred from Bowen et al. [35], p. 8

*Acceptability* indicates how the intended participants and program staff react to the intervention [35]. Regarding the educational materials in this study, a woman shared, “The picture is good and makes it easy for us to understand.” Another woman mentioned, “We are very happy to learn many things, no one feels bad. Everybody is happy.” After the education, a woman said, “Husbands should change their behaviors. For example, giving the women heavy loads.” One man stated, “We learned how to make a good relationship between pregnant women and their families.” Another man expressed, “We learned to increase love to unborn baby and his/her mother.”

*Demand* indicates the estimated or actual use of the intervention. As an example of demand, a man stated, “We need to tell the government to add more health centers to the villages near people’s houses.” Another man mentioned, “This type of education should be given to all Tanzanian people, pregnant women, and their families.”

*Implementation* indicates the extent, likelihood, and manner in which an intervention is fully implemented. In this study, more time was needed to complete the self-administered questionnaire than as planned.

*Practicality* indicates the extent to which an intervention can be delivered depending on the available resources, time, commitment, or a combination of factors. We identified that a local collaborator can conduct the research; however, training is required to be able to extend the research.

*Limited efficacy* indicates whether the study was successful even at a limited extent such as the evaluation of a convenient sample. We conducted this study using a convenient sample and we found several statistically significant findings. If we conduct this study using a larger sample with possible randomization, it is expected that more significant differences will be found after the intervention.

## Discussion

The *family-oriented antenatal group educational program* in rural Tanzania succeeded in providing information about the importance of birth preparation and how to strengthen family involvement. The program specifically improved the scores of *Knowledge*, *Family Support,* and *Preparation of Money and Food* among all the participants. As *Knowledge* increased among all the participants, the educational program utilizing a picture drama and discussion was effective and culturally compatible with the participants.

It was an important finding that *Family Support* changed significantly. This variable includes the items about discussion on birthplace among family members. The increase of discussion among family members including women may improve women’s decision-making power within the family. Household equity indeed has also been shown to increase women’s facility delivery in Nepal [36]. Notably, when Nepalese women were actually communicating with their husbands, they were more likely to discuss health issues during pregnancy and birth preparedness with their husbands, and the husbands had a higher likelihood of being present during health facility delivery [37]. When women in Guatemala [38] and Uganda [39] made a birth plan with their husbands or other family members, they were more likely to seek the care of SBAs. Thus, although we did not increase SBA-assisted deliveries this time, the educational program contributed to increasing the participation of women in decision-making and birth preparation, which could increase facility delivery with a larger sample size.

Similarly, the increase of monetary preparation from the program shows that the program had the potential to increase SBA-assisted deliveries. Other studies also showed that monetary preparation was found to be one of the key factors for achieving deliveries with SBAs [8, 33, 40].

The next step is the necessity to improve education on *Preference of SBA*. Among pregnant women, the score was significantly decreased; this change was not our intent. This factor included perceptions toward health care providers. We deduced that the participants from this rural area might have had perceptions toward health care providers that the educational program did not address. For example, disrespect and abuse of birthing women by health care providers have been reported in Tanzania [41–43]. As suggested by WHO [3], education for health care providers might also be needed. Another possible area for study is a follow-up of community volunteers for women to utilize skilled care at delivery as recommended by a *community-based safe motherhood program* in Tanzania [44]. As the present study provided only 1 session of education, continuous education or follow-up might be a more effective method for increasing readiness to use a health facility for an emergency.

Regarding the changes in the scores between family members and pregnant women, family members showed an increase in the scores for more variables than pregnant women. For example, the scores for *Birth Preparedness* and *Avoidance of Medical Intervention* increased significantly among only the family members. Some possible explanations why the scores of pregnant women did not significantly increase were the higher pre-test scores and the smaller sample size of the pregnant women. On the other hand, the scores for the *Home-based Value* of women decreased significantly, which was not our intended change. As the factor was composed of reverse items, the participants might not have answered carefully and instead answered in a response set, which is the opposite course. Reverse items could be reduced so that participants answer the questionnaires as researchers intended.

The study was significant in terms of obtaining preliminary data from pregnant women and their families who were difficult to reach in rural Tanzania. The study can be further improved in terms of more efficient implementation and greater community involvement in a subsequent larger scale study. At this stage of the educational program development, the study limitations of a small sample size, less rigorous sampling methods, and no-comparison group restrict the demonstration of the robust effects of the educational program, and therefore the generalizability is limited. Due to the small sample size, normality was not confirmed, and therefore only non-parametric analysis was used. With a larger sample size, the BPQ could benefit from additional psychometric testing to clarify and strengthen the concepts. In addition, this study would benefit more if it were conducted in districts with higher home birth rate. Although this study focused on increasing the knowledge and psychological aspects of BP/CR immediately after education, it did not evaluate outcome behaviors, such as delivery at a health facility. Further study is needed to investigate whether the participants who received education delivered with SBAs at a health facility, and to clarify the neonatal and maternal outcomes. Ultimately, a longitudinal randomized controlled study that replicates the educational intervention and includes outcome data in rural settings of Tanzania will provide more generalizable data.

## Conclusions

The *family-oriented antenatal group educational program* has potential to increase knowledge, birth preparedness, and awareness of the need for family support among pregnant women and their families in rural Tanzania. As the contents of the program can be taught easily by reading the picture drama, lay personnel, such as community health workers or traditional birth attendants, can use it in villages. Further development of the Birth Preparedness Questionnaire is necessary to strengthen the involved factors. A larger scale study with a more robust BPQ and documentation of skilled care use is needed for the next step.

## Abbreviations

BP/CR: Birth Preparedness and Complication Readiness
BPQ: Birth Preparedness Questionnaire
MDGs: Millennium Development Goals
SBAs: Skilled Birth Attendants
WHO: World Health Organization
